# Low feed dose-to-larval density attenuates pathogen inactivation in black soldier fly larvae bioconversion

**DOI:** 10.3389/fmicb.2026.1909800

**Published:** 2026-09-23

**Authors:** Evans Were, Björn Vinnerås, Cecilia Lalander

**Affiliations:** Department of Energy and Technology, Swedish University of Agricultural Sciences, Uppsala, Sweden

**Keywords:** biowaste treatment, circular economy, *Escherichia coli*, food safety, *Hermetia illucens*, One Health, resource recovery, *Salmonella*

## Abstract

Black soldier fly, *Hermetia illucens* larvae (BSFL) convert biowaste substrates into high-value products and can reduce notorious food-borne pathogens during the bioconversion process. Substrate properties, feed dose, and larval density are key factors that influence process efficiency, yet their effect on pathogen reduction is unclear. We addressed this gap in laboratory-scale experiments using BSFL reared at different larval densities (0, 1, 3 and 6 larvae cm^–2^) with a corresponding feed dose of 0.6, 0.2 and 0.1 g VS larva^–1^, respectively. This resulted in larval density and area adjusted feed dose, or *feed dose-to-larval density*, of 2.6, 0.9 and 0.4 mg VS larva^–1^ cm^–2^. BSFL were fed a carbohydrate-rich or protein-rich substrate supplied in three feeding events. Pathogen inactivation was assessed in substrates inoculated with a cocktail of bacterial pathogens (*Salmonella* spp. and *Escherichia coli*) either on the first feeding event (single inoculation), or on all three feeding events (multiple inoculations). Low feed dose-to-larval density enhanced bioconversion efficiency, larval yield and substrate reduction, but reduced larval survival, final larval weight and frass yield. Bioconversion efficiency and substrate reduction were higher in the carbohydrate-rich than the protein-rich substrate. Conversely, pathogen reduction (Δlog_10_Red) was higher in the protein-rich than carbohydrate-rich substrate. Unexpectedly, Δlog_10_ Red declined with decreasing feed dose-to-larval density, with multiple substrate inoculations causing greater decline than single substrate inoculation. Collectively, our findings suggest that process efficiency and pathogen inactivation are inextricably linked: low feed dose-to-larval density enhances process efficiency but could compromise microbiological hygiene of the process and products. This trade-off underscores a need for integrated optimization of process efficiency and hygiene in BSFL bioconversion.

## 1 Introduction

The black soldier fly, *Hermetia illucens* L. (Diptera: Stratiomyidae) is a holometabolous insect whose larval stage (BSFL) exhibits polyphagous and saprophagous lifestyles (Nguyen et al., 2015). BSFL can be reared on a wide range of biowastes including agro-industrial by-products, abattoir waste, food waste, and manure (Nguyen et al., 2015; Lalander et al., 2019; Lopes et al., 2023). During bioconversion, BSFL consume and convert the nutrients in biowaste substrates into two key high-value products: nutrient-rich larval biomass (used in feed formulations) and a treatment residue called frass (used mainly as a biofertilizer) (Beesigamukama et al., 2021; Lopes et al., 2022). Thus, BSFL bioconversion is an emerging technology for treatment of biowaste with potential to provide a closed-loop approach for advancing global sustainability initiatives such as a circular economy and One Health (Kasima et al., 2025; Bruno et al., 2025; Barragán-Fonseca and Gómez, 2025). A key benefit of BSFL bioconversion is the inactivation or reduction of notorious food-borne pathogens such as *Escherichia coli*, *Salmonella* spp., and *Staphylococcus aureus* when present in the substrates (Erickson et al., 2004; Lalander et al., 2015; Lopes et al., 2020; Gorrens et al., 2021; Zhang et al., 2022; Lalander et al., 2025). Pathogen inactivation is crucial for the hygienic safety of the bioconversion process and products, yet the mechanism of pathogen inactivation remains unclear.

Diseases caused by food-borne pathogens such as pathogenic *E. coli* and *Salmonella* spp. remain a major public health concern and economic burden globally (Ikuta et al., 2022). For example, *Salmonella enterica* subsp. *enterica* serovar Typhi (*S.* Typhi) is a human-restricted pathogen that causes about 9⋅3 million cases of typhoid fever and over 100,000 deaths annually (Murthy et al., 2025). Conversely, the non-typhoidal serovar *Salmonella enterica* subsp. *enterica* serovar Dublin (*S*. Dublin) is host-adapted to cattle (Pharo et al., 2025). Outbreaks of *S.* Dublin can lead to high mortality rates particularly in calves (Pharo et al., 2025). *Escherichia coli* (all pathotypes combined) was associated with about 950,000 human deaths in 2019 and ranked second among the top five bacterial pathogens, with the highest burden in Sub-Saharan Africa and Asia (Ikuta et al., 2022). Outbreaks of food-borne pathogens are primarily associated with the ingestion of contaminated food or feed (Murthy et al., 2025).

In a batch system, BSFL treatment of human feces achieved a 6 log_10_ reduction of *S.* Typhimurium within 8 days, compared to less than 2 log_10_ in controls (Lalander et al., 2013). Comparably, in a continuous system, BSFL treatment reduced *S*. Typhimurium from about 7 log_10_ in the substrate to about 4 log_10_ in frass within 2 weeks, and below detection limit after 7 weeks (Lalander et al., 2015). Pathogen reduction in BSFL bioconversion is inextricably linked to factors that impact process efficiency. For example, apportioning the substrate into three feeding events enhanced process efficiency and reduced *Salmonella* spp. and *E. coli* ATCC 13706 during BSFL bioconversion of bread-aquaculture waste mixture (Lopes et al., 2020). Notably, pathogen reduction was pronounced when the substrate was inoculated only on the first feeding event (Lopes et al., 2020).

The type of substrate can also influence pathogen inactivation. For example, Erickson et al. (2004) reported accelerated reduction of *S.* Enteritidis ME18 and *E. coli* O157:H7 in BSFL- treated chicken manure. Compellingly, no pathogen reduction was observed in cow or hog manure, although the latter promoted larval growth by over two-fold compared to chicken manure. By contrast, Liu et al. (2008) observed inactivation of *E. coli* ER2566 in BSFL- treated dairy cow manure. While this incongruity was largely attributed to the differences in *E. coli* strains used, other factors may influence pathogen inactivation, which if well understood, could guide simultaneous optimization of process efficiency and microbiological hygiene in BSFL bioconversion.

Process efficiency of BSFL bioconversion is assessed using several process parameters including substrate-to-biomass conversion efficiency (bioconversion efficiency), larval yield, larval survival and substrate reduction (Lalander et al., 2019). Larval density (larvae cm^–2^) is another factor that has been shown to impact process efficiency. Generally, high larval density is associated with reduced individual final larval weight and size, regardless of substrate type (Barragán-Fonseca et al., 2018; Schreven et al., 2022; Lopes et al., 2023; Muurmann et al., 2025; Kharel et al., 2026; Parra Paz et al., 2015; Nayak and Klüber, 2025). High larval density has also been reported to upregulate immune responses in BSFL. Opare et al. (2023) revealed that BSFL reared at a high density (10 larvae cm^–2^) exhibited elevated activity of the enzyme phenoloxidase, compared to BSFL reared at 5 and 1 larva cm^–2^. As a key component of humoral immunity, phenoloxidase catalyzes the melanization reaction, which is conserved in insects (Cerenius et al., 2008). High population density in insects can also trigger constitutive upregulation of immune defenses, including the secretion of antimicrobial peptides (AMPs), due to a perceived risk of infection (Wen et al., 2025). This biological phenomenon, termed density-dependent prophylaxis has been reported in various insects including desert locusts (*Schistocerca gregaria*), leaf-cutting ants (*Acromyrmex echinatior*), and the cotton leafworm (*Spodoptera lituralis*) (Hughes et al., 2002; Wilson et al., 2002; Cotter et al., 2004; Wen et al., 2025).

Constitutive immune responses are nutritionally and energetically costly (Britton and Davidowitz, 2025). Lee et al. (2006) showed that dietary protein exerts stronger effects on constitutive immune responses and pathogen resistance than dietary carbohydrate. Similarly, using transcriptome analysis, Vogel et al. (2018) found that the expression of AMP-encoding genes was higher in BSFL reared on protein-rich substrates. AMP biosynthesis by BSFL is, in part, linked to pathogen inactivation during bioconversion, and conditions that promote AMP biosynthesis could enhance pathogen inactivation (Xia et al., 2021; Liu et al., 2024). However, while macronutrient (e.g., protein and carbohydrate) composition of substrates and larval density are known to impact process efficiency, their impact on pathogen reduction is unclear (Barragan-Fonseca et al., 2019; Lalander et al., 2019; Lopes et al., 2023; Muurmann et al., 2025). We hypothesized that high larval density with a decreasing feed dose (*i.e.*, a low feed dose-to-larval density) promotes pathogen inactivation, particularly in protein-rich substrates where dietary protein supports constitutive larval immunity responses and larval growth. Accordingly, the specific objectives of this study were to assess the effect of feed dose-to-larval density and substrate type (protein- and carbohydrate-rich) on (i) process efficiency and (ii) the dynamics and inactivation of bacterial pathogens (*Salmonella* spp. and *E. coli*) under single and multiple substrate inoculations in BSFL bioconversion.

## 2 Materials and methods

### 2.1 Chemicals, microbial strains and culture conditions

All chemicals, unless otherwise stated, were purchased from VWR (Stockholm, Sweden). Six bacterial strains were used in the pathogen inactivation experiments: two strains of *E. coli* (ATCC 8739 and ATCC 13706) and four serotypes of *Salmonella enterica* (*S.* Abony, *S.* Dublin, *S.* Senftenberg, and *S*. Typhimurium). Cultures of each isolate were obtained by streaking from cryo-preserved stocks. Horse blood agar (SVA, Uppsala Sweden) was used for *E. coli*. Xylose lysine desoxycholate (XLD) agar containing 0.15 % sodium-novobiocin (SVA) was used for *Salmonella* spp. Cultures were incubated at 37 °C overnight in an incubator (Memmert GmbH, Germany). Inoculum was prepared from the fresh cultures using nutrient broth (Oxoid AB, Sweden) as described in Lopes et al. (2020).

### 2.2 Seed larvae and substrates

Seed larvae (about 5 days old and 1.8 mg larva^–1^) were obtained from an established colony of *H. illucens*. The colony has been operating since 2015 at the Department of Energy and Technology, Swedish University of Agricultural Sciences (SLU). Larvae were separated from the residues by sieving through layers of sieves (Retsch GmbH, Germany). Larvae retained between a 0.6- and 2.0 mm sieve were taken as seed larvae. From these, three batches of 0.1–0.3 g of larvae were weighed, manually counted and used for the calculation of larval density. Triplicate samples of seed larvae and substrates were also taken for physico-chemical analysis (described in Section 2.6). Two contrasting substrates (a carbohydrate-rich and protein-rich) were used in this study. The carbohydrate-rich substrate consisted of a mixture of bread waste (40 % w/w) and cabbage waste (60 % w/w). Cabbage waste was obtained from a vegetable wholesaler (Grönskashallen Sorunda, Stockholm, Sweden). Bread waste (pre-packed loaves and buns from various bakeries across Sweden) was donated by a local supermarket in Uppsala. The protein-rich substrate consisted of a mixture of brewers spent grains (60 % w/w), pig abattoir waste (fresh abdominal and thoracic organs; 40 % w/w) and bovine blood (5 % v/w). Abattoir waste was obtained from Lövsta Experimental farm, SLU, Uppsala. Pig production at Lövsta is certified specific-pathogen-free. Thus, the waste poses no risk of infectious agents. Brewers spent grains were obtained from a local brewery in Knivsta, Sweden. To prepare the substrates, the waste streams were separately milled using a centrifugal mill (Voran Maschinen GmbH, Austria), mixed on wet weight basis and stored at −20 °C until use.

### 2.3 Experimental setup

Three main experiments were conducted in this study (Figure 1). The treatments investigated were two contrasting substrate types (a carbohydrate-rich and protein-rich substrate) and three larval densities (1, 3 and 6 larvae cm^–2^). As the same amount of substrate was provided to BSFL, the larval feed dose at each larval density was 0.6, 0.2 and 0.1 g VS larva^–1^, respectively. The combined effect of feed availability and larval density was captured by a density- and area-adjusted feed dose (feed dose-to-larval density factor), calculated as total VS divided by the product of larval number and rearing area. This factor was 2.6, 0.9 and 0.4 mg VS larva^–1^ cm^–2^, respectively. The impact of treatments on process efficiency was assessed in Experiment 1. Experiments 2 and 3 were conducted to assess the impact of treatments on pathogen dynamics and inactivation. Treatments were performed in 231 cm^2^ dark plastic containers (21 L × 17 W × 11 H -cm; SmartStore; Orthex Sweden AB, Sweden). Treatments were conducted in triplicates and experiments were independently repeated at least twice.

**FIGURE 1:**
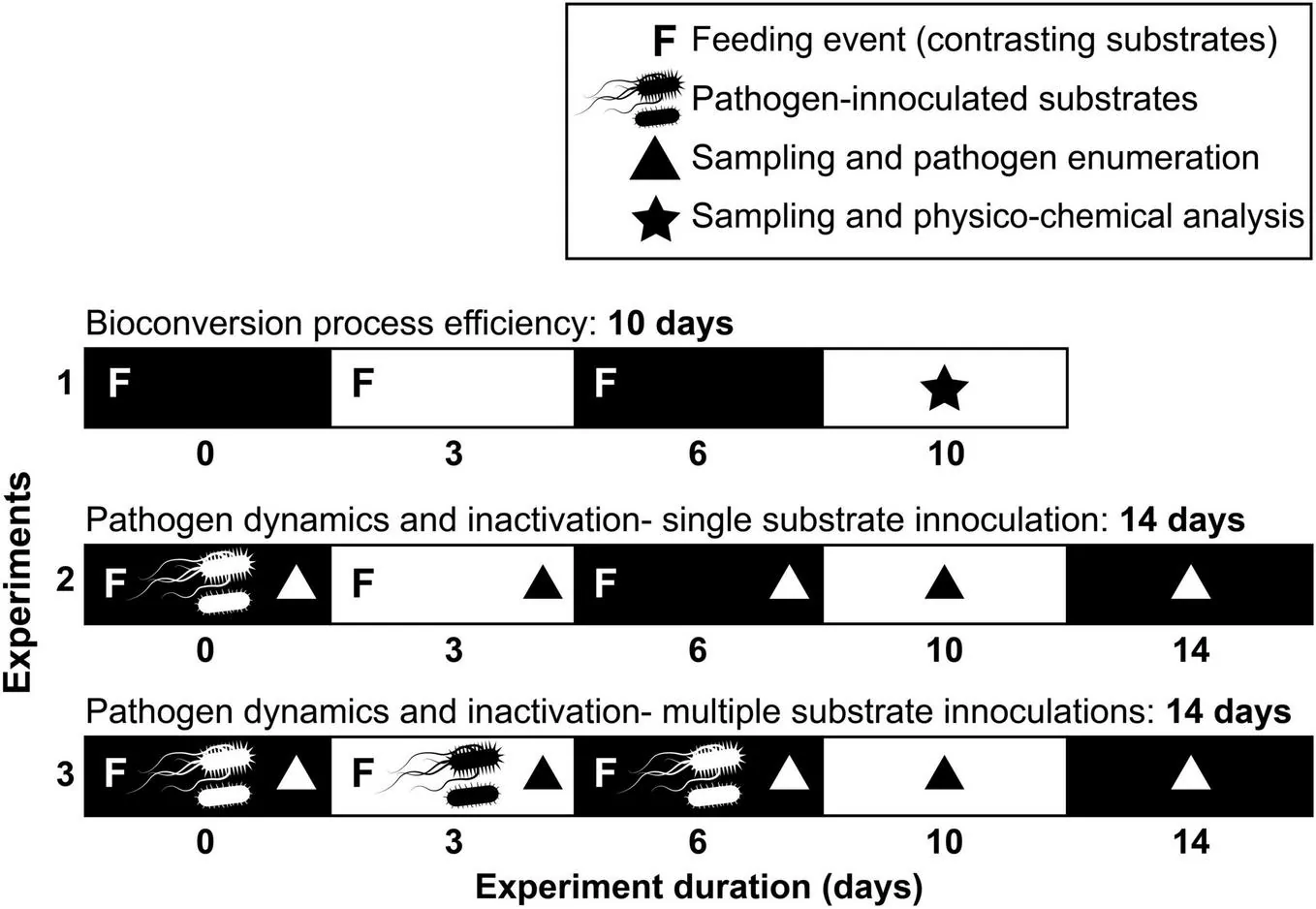
Schematic overview of the study. Black soldier fly, *Hermetia illucens* larvae (BSFL) were reared at three feed dose-to-larval densities (2.6, 0.9 and 0.4 mg VS larva^–1^ cm^–2^). The impact of treatments on process efficiency was assessed in Experiment 1. The impact of treatments on the dynamics and inactivation of bacterial pathogens (*Salmonella* spp. and *Escherichia coli*) were investigated in over a period of 14 days in Experiments 2 and 3. Experiments 2 and 3 followed single or multiple substrate inoculations, respectively. Samples were taken at selected time points for pathogen enumeration and physico-chemical analysis.

### 2.4 Bioconversion process efficiency

Treatments were performed in dark plastic containers (21 L × 17 W × 11 H- cm; SmartStore). Substrates were added to each container, followed by a total of 231, 693, or 1386 seed larvae to achieve larval densities of 1, 3 and 6 larvae cm^–2^, respectively. Controls without larvae were also included. For randomization, containers with treatments were placed into a larger grey container (60 L × 40 W × 12 H -cm; Auer Packaging, Germany). The larger containers were then loaded onto a bakery trolley (ToFo Trading AB, Sweden) and incubated for 10 days in the dark under temperature regulated to 30 ± 3 °C to allow bioconversion. During this period, larvae were fed by addition of fresh substrate to each treatment container. Feeding was performed on three events (Day 0, 3 and 6; Figure 1). After each feeding event, the small and larger containers were randomized again by alternation of their positions. After 10 days, the experiment was terminated and samples of larvae and frass taken for physico-chemical analysis. Samples were stored at −20 °C until used for physico-chemical analysis (described in Section 2.6).

### 2.5 Pathogen dynamics and inactivation

Assessment of pathogen dynamics and inactivation (Experiments 2 and 3; Figure 1) was conducted in a Biosafety Level 2 laboratory. The treatments and experimental setup of Experiment 2 were similar to Experiment 1, with some notable modifications. Firstly, in Experiment 2, BSFL were fed pathogen-inoculated substrates only on the first feeding event (*i.e.*, single substrate inoculation). In the subsequent two feeding events (Day 3 and 6), BSFL were fed non-inoculated substrates. Secondly, the duration of Experiment 2 was 14 days. For Experiment 3, the setup and treatments were identical to those in Experiment 2, except that BSFL were fed pathogen-inoculated substrates at each of the three feeding events (Day 0, 3 and 6; *i.e.*, multiple substrate inoculations; Figure 1). Freshly prepared bacterial inoculum was used for substrate inoculation as described in Lopes et al. (2020). Substrates were inoculated with the bacterial suspension (1 % v/w) and gently homogenized using a sterile spatula. Inoculated substrates were added to treatment containers followed by the seed-larvae. Containers were randomized as described earlier and incubated for 14 days at 30 ± 3 °C in a Hydro Shoot 120 tent (Secret Jardin-Agomoon, Belgium). Temperature in the tent was regulated using a self-regulating heater (Trixie, Germany).

At selected time points (Figure 1), 5 g of frass was weighed into 50-mL Falcon tubes for simultaneous enumeration of *Salmonella* spp. and *E. coli* following the procedure described in Lopes et al. (2020). Briefly, the sample was suspended in 45 mL of buffered peptone water containing 0.1 % (v/v) Tween 80 (pH 7.0; Merck, Germany) hereafter referred to as Tween buffer. To do this, tubes were vortexed for 1 min to suspend the sample. After 20 min of repose, tubes were vortexed again and this homogenate was regarded as dilution 1, from which additional ten-fold serial dilutions were prepared using Tween buffer. Aliquots (0.1 mL) from selected dilutions were spread onto selective culture media: Chromocult agar (MicLev AB, Sweden) for the enumeration of *E. coli* or XLD agar (SVA) for the enumeration of *Salmonella* spp. Plates were incubated at 37 °C in an incubator for 24 h after which pathogen colonies were enumerated and expressed as log_10_ CFU g^–1^.

### 2.6 Physico-chemical analyses

Amino acid composition in the substrates was determined according to the standard method for sampling and analytical methods for inspection of feed (European Commission (EC) regulation 152/2009). For this purpose, 50 g of fresh substrate sample was lyophilized using a freeze dryer (Mechatech Systems, UK). Samples were pulverized using a mortar and pestle and analysed at the Core Facility, University of Hohenheim, Germany. Amino acid composition was determined following Commission Regulation (EC) No 152/2009. Additionally, proximate analysis was conducted by an accredited commercial laboratory (Eurofins Food & Feed Testing, Sweden).

Analysis of total solids (TS) and volatile solids (VS) was conducted on samples obtained from fresh substrates, seed-larvae, as well as harvested larvae and frass obtained at the end of the experiments. For determination of TS, pre-weighed samples were dried at 65 °C for 48 h and re-weighed thereafter (Vahlberg et al., 2013). For determination of VS, the dried samples were combusted at 250 °C for 2 h followed by 550 °C for 4 h in muffle furnace (Nabertherm GmbH, Germany). To determine the pH of fresh substrates and frass, 5 g of sample was weighed into a 50-mL Falcon tube and suspended in 20 mL of deionised water by vortexing for 1 min. After 1 h of repose, tubes were vortexed again and the pH was determined using a pH meter (S210 SevenCompact, Mettler Toledo, Switzerland).

### 2.7 Calculations

Data from Experiment 1 was used to determine process efficiency, by calculating process parameters. Process parameters including bioconversion efficiency, larval yield, and substrate reduction were calculated using standard equations. Bioconversion efficiency (%) was calculated on dry matter (TS basis) using Equation 1:


Bioconversion⁢efficiency=(TSl⁢a⁢r⁢v⁢a⁢emTSi⁢n⁢i⁢t⁢i⁢a⁢l⁢s⁢u⁢b⁢s⁢t⁢r⁢a⁢t⁢em)×100 (1)

where *_*m*_*TS*_*larvae*_* and _*m*_TS_*initial substrate*_ are the total mass of total solids in the harvested larvae and the initial substrate, respectively. Similarly, material reduction (%) was calculated on dry matter (TS basis) using Equation 2:


Substrate⁢reduction=(1-TSf⁢r⁢a⁢s⁢smTSi⁢n⁢i⁢t⁢i⁢a⁢l⁢s⁢u⁢b⁢s⁢t⁢r⁢a⁢t⁢em)×100 (2)

where _*m*_TS_*frass*_ and _*m*_TS_*initial substrate*_ are the total mass of total solids in the frass and the initial substrate, respectively. Larvae survival (%) was calculated using Equation 3:


Larvae⁢survival=larvaee⁢n⁢dnlarvaes⁢t⁢a⁢r⁢tn×100 (3)

where *_*n*_*larvae_*end*_ and *_*n*_*larvae_*start*_ are the total number of larvae at the end and at the start of the experiment, respectively.

Pathogen inactivation was determined using data from Experiment 2 and 3, by calculating three parameters: *inactivation rate constant* (*k*), which represents the log_10_ reduction per time unit; *decimal reduction time*, which represents the time (in days) for achieving 1 log_10_ reduction (90 % reduction in the concentration); and the total logarithmic pathogen reduction (ΔlogRed), which reflects the total reduction of a selected pathogen from initial to final concentration. Total logarithmic pathogen reduction was calculated using Equation 4:


$$\Delta \,\text{logRed}={\text{log}}_{10}\,\left(\frac{{\text{[Pathogen]}}_{t=0}}{{\text{[Pathogen]}}_{t=i}}\right)$$ (4)

where [Pathogen]_*t* = 0_ is the initial pathogen concentration (CFU g^–1^) of a selected pathogen at start of the experiment and [Pathogen]_*t* = *i*_ is the final concentration of the same pathogen at time *i*. The inactivation rate constant, *k* (log_10_ reduction per day) was calculated using Equation 5:


$$k=\frac{\log_{10}\,N_{\mathrm{ti}}-\log_{10}\,N_{\mathrm{t0}}}{t_{i}-t_{0}}$$ (5)

where N_*t*_ and N_*0*_ are the pathogen concentration (CFU g^–1^) at time (i) t_*i*_ (in days) and at the start of the experiment (t_*0*_), respectively. The decimal reduction time (D_90_), was calculated using Equation 6:


$${\text{D}}_{90}=\frac{-1}{k}$$ (6)

Four *Salmonella enterica* serovars were inoculated and enumerated collectively and the reported inactivation kinetics thus represent the behaviour of the *Salmonella* spp. cocktail and do not resolve potential serovar-specific differences. The choice to include more than one *Salmonella* serovar was made to increase the robustness of the results and reduce their dependence on the sensitivity of a single serovar, as different *Salmonella* serovars have been shown to vary in their sensitivity to fly larvae bioconversion (Gabler, 2014). Calculations for pathogen inactivation in Experiment 3 were based on the final feeding event.

### 2.8 Statistical analyses and data visualization

Statistical analysis and visualizations were performed using R (v.4.4.2; R Core Team, 2025) in R studio environment (v. 2023.03.1). Prior to statistical analysis, data was checked for normality and homoscedasticity at a 5 % significance level (Kozak and Piepho, 2018). Homoscedasticity of the data was verified using Levene’s test, whereas the normality of the data was verified using the Shapiro-Wilk test and diagnostic plots (histograms and Q-Q plots). In cases where the assumptions for parametric tests were met, analysis of variance (ANOVA) and subsequent *post hoc* analysis using Tukey’s Honest Significant Difference (Tukey HSD) test were used at a 5 % significance level to test significant differences between the means. In Experiment 2, the data for D_90_ value of *Salmonella* spp. and *E. coli*, and the *k* value of *E. coli* were non-parametric. Similarly, in Experiment 3, the data for D_90_ value and the *k* value for both pathogens were non-parametric. Therefore, this data was analysed using the Scheirer-Ray-Hare test (an extension of the Kruskal-Wallis test) at 5 % significance. A subsequent *post hoc* Dunn’s test was conducted to assess all pairwise of comparisons among groups using the Benjamini-Hochberg adjustment for multiple testing (adjusted *p*-value) (Benjamini and Hochberg, 1995). Where applicable, comparisons were made between treatments and the control. Unless otherwise stated, all values represent mean ± standard deviation.

## 3 Results

### 3.1 Substrate physico-chemical properties

Most substrate physico-chemical properties, except nitrogen-free extract (NFE), were two-to-three times higher in the protein-rich than the carbohydrate-rich substrate (Table 1). For example, the crude protein composition of protein-rich substrate was 32 % of DM (dry matter) compared to 15 % in the carbohydrate-rich substrate. In contrast, NFE (a proxy for non-fibrous, soluble and more readily digestible carbohydrates) was higher in the carbohydrate-rich substrate (68 % of DM) than the protein-rich substrate (32 % of DM). The carbohydrate- and protein-rich substrates moisture content of 74 and 76 %, respectively. Other properties such as pH, dry matter content, VS and ash were similar between substrates (Table 1).

**TABLE 1:** Physico-chemical and nutritional properties of the carbohydrate- and protein-rich substrates used in this study.

Substrate properties	Carbohydrate-rich	Protein-rich
Essential amino acids (mg g–1) DW		
Histidine	3.0 ± 0.1	8.1 ± 0.2
Isoleucine	5.1 ± 0.2	11.1 ± 0.4
Leucine	9.8 ± 0.3	24.5 ± 0.4
Lysine	4.1 ± 0.1	19.6 ± 0.3
Methionine	2.1 ± 0.1	6.1 ± 0.1
Phenylalanine	6.8 ± 0.1	14.3 ± 0.4
Threonine	4.9 ± 0.0	12.9 ± 0.2
Tryptophan	1.6 ± 0.1	3.9 ± 0.2
Valine	5.1 ± 0.2	14.9 ± 0.6
Non-essential amino acids (mg g–1) DW		
Alanine	5.6 ± 0.1	17.7 ± 0.5
Aspartic Acid	8.5 ± 0.1	24.2 ± 0.4
Glutamic Acid	43.3 ± 0.9	40.5 ± 0.8
Serine	7.7 ± 0.1	12.5 ± 0.2
Glycine	5.7 ± 0.1	16.1 ± 0.7
Proline	14.2 ± 0.3	17.9 ± 0.9
Arginine	5.7 ± 0.2	13.6 ± 0.3
Cysteine	3.0 ± 0.1	4.0 ± 0.1
Tyrosine	4.1 ± 0.2	7.7 ± 0.4
Physicochemical and nutritional properties		
pH	5.1 ± 0.5	5.4 ± 0.6
Dry matter (DM) content (%)	26.1 ± 2.1	24.0 ± 0.6
Total volatiles solids (VS) (% of DM)	95.9 ± 0.7	95.6 ± 0.1
Nitrogen-Free Extract, NFE (% of DM)	67.9 ± 0.6	32.2 ± 1.9
Crude protein (% of DM)	15.4 ± 0.4	31.9 ± 0.8
Crude fat (% of DM)	4.6 ± 0.1	11.6 ± 1.2
Crude fibre (% of DM)	4.4 ± 0.2	9.2 ± 0.4
Ash (% of DM)	3.7 ± 0.0	4.3 ± 0.0

All values represent mean ± standard deviation.

### 3.2 Experiment 1: process efficiency

Bioconversion efficiency increased significantly with decreasing feed dose-to-larval density (*F*_2,66_ = 124.5, *P* < 0.001; Figure 2a). Moreover, bioconversion efficiency was significantly impacted by substrate type (*F*_1,66_ = 15.1, *P* < 0.001), but not the interaction of feed dose-to-larval density and substrate type (*F*_1,66_ = 0.85, *P* = 0.433). At 2.6 mg VS larva^–1^ cm^–2^, bioconversion efficiency was 15.1 ± 2.2 % but increased by nearly two-fold reaching 28.2 ± 3.72 % at 0.9 mg VS larva^–1^ cm^–2^ in the carbohydrate-rich substrate (Figure 2a). A similar pattern was observed in the protein-rich substrate (Figure 2a). Larval yield increased significantly with decreasing feed dose-to-larval density (*F*_2,66_ = 387.7, *P* < 0.001), but was similar between substrates (*F*_1,66_ = 2.2, *P* = 0.139; Figure 2b). Additionally, a significant interaction effect on larval yield was observed between feed dose-to-larval density and substrate type (*F*_2,66_ = 5.0, *P* < 0.001).

**FIGURE 2:**
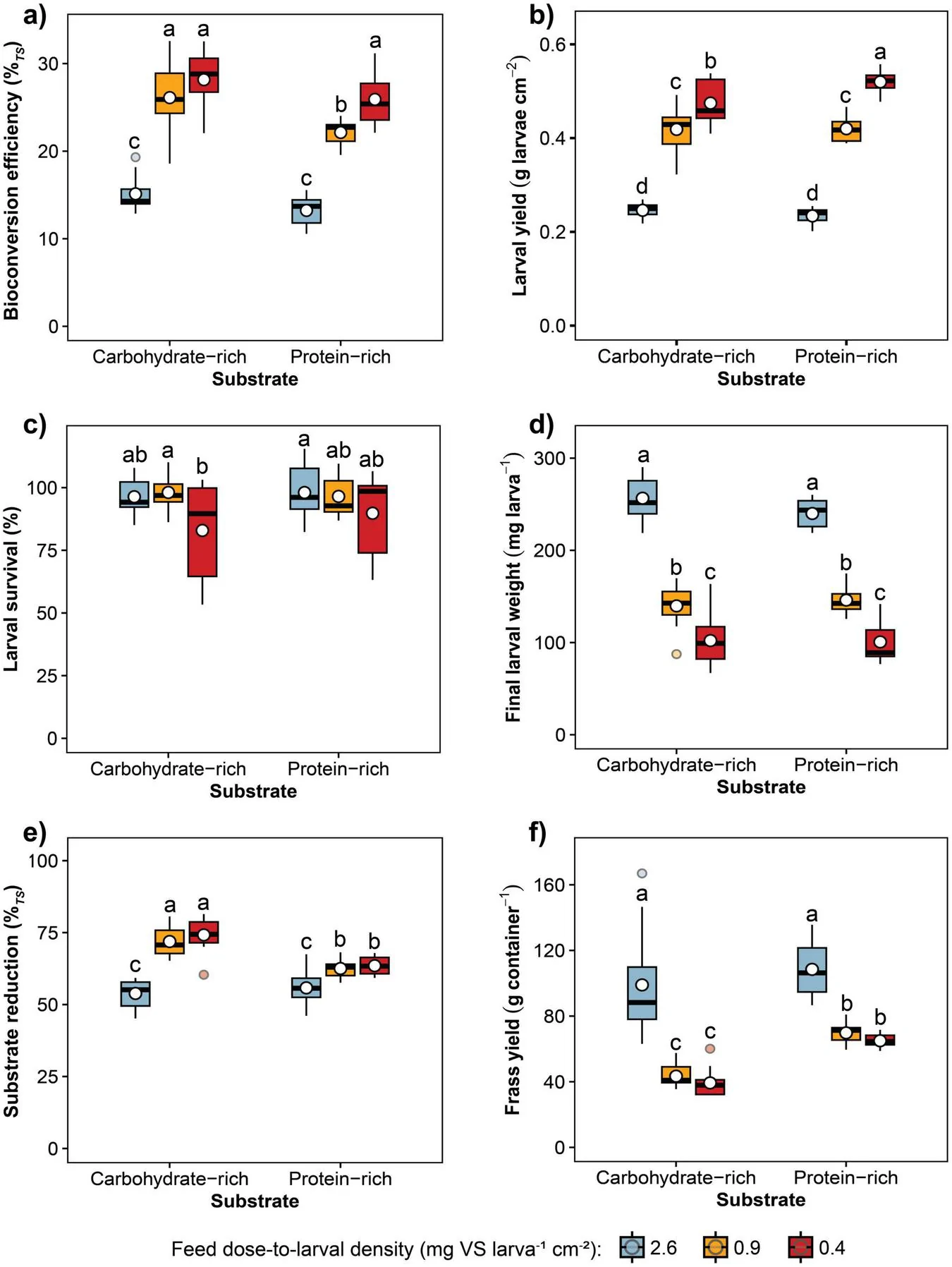
Boxplots summarizing process efficiency parameters after bioconversion of the carbohydrate-rich and protein-rich substrate in Experiment 1 using black soldier fly, *Hermetia illucens* larvae (BSFL): **(a)** bioconversion efficiency (% TS), **(b)** larval yield per treatment (g larvae cm^–2^), **(c)** larval survival (%), **(d)** final larval fresh weight (mg larva^–1^), **(e)** substrate reduction (% TS), and **(f)** frass yield per treatment. Different letters indicate statistically significant differences among treatments. Boxes display interquartile range (IQR), median (bold horizontal line inside each box), mean (white circle), and the range of data represented by whiskers (vertical lines). Data points farther than 1.5 × IQR from boxes denote outliers.

Larval survival declined significantly with decreasing feed dose-to-larval density (*F*_2,66_ = 6.0, *P* < 0.001), but was not impacted by substrate type (*F*_1,66_ = 0.6, *P* > 0.05) or the interaction between feed dose-to-larval density and substrate type (*F*_2,66_ = 0.7, *P* > 0.05; Figure 2c). Generally, larval survival exceeded 80 % across all treatments but was highest at 2.6 mg VS larva^–1^ cm^–2^, reaching 96.4 ± 7.3 % and 98.0 ± 11.1 % in carbohydrate- and protein-rich substrates, respectively (Figure 2c). However, survival declined to 82.9 ± 19.3 % and 89.8 ± 17.1 % in the lowest feed dose-to-larval density (0.4 mg VS larva^–1^ cm^–2^) in the carbohydrate-rich and protein-rich substrate, respectively (Figure 2c). Final larval weight declined significantly with decreasing feed dose-to-larval density (*F*_2,66_ = 281.7, *P* < 0.001), but was similar between substrates (*F*_1,66_ = 0.6, *P* > 0.05; Figure 2d and Supplementary Figure 1). Moreover, no significant interaction effect on final larval weight was observed between feed dose-to-larval density and substrate type (*F*_2,66_ = 1.7, *P* > 0.05). At the highest feed dose-to-larval density (2.6 mg VS larva^–1^ cm^–2^), BSFL exhibited the highest final larval weight, reaching 256.6 ± 23.6 mg and 240 ± 15.9 mg larva^–1^ in the carbohydrate-rich and protein-rich substrate, respectively (Figure 2d). By contrast, final larval weight declined by nearly two-fold, reaching 102.1 ± 28.2 mg and 100.6 ± 24.4 mg larva^–1^ at the lowest feed dose-to-larval density in carbohydrate-rich and protein-rich substrates, respectively.

Substrate reduction increased significantly with decreasing feed dose-to-larval density (*F*_2,66_ = 57.9, *P* < 0.001) but was generally higher in the carbohydrate-rich than the protein-rich substrate (*F*_1,66_ = 27.0, *P* < 0.001; Figure 2e). Additionally, a significant interaction effect on substrate reduction was also observed between feed dose-to-larval density and substrate type (*F*_2,66_ = 11.9, *P* < 0.001; Figure 2e). By contrast, frass yield declined with decreasing feed dose-to-larval density (*F*_2,66_ = 73.9, *P* < 0.001) and was higher in the protein-rich than carbohydrate-rich substrate (*F*_1,66_ = 28.6, *P* < 0.001; Figure 2f). No significant interaction effect on frass yield was observed between substrate type and feed dose-to-larval density (*F*_2,66_ = 2.07, *P* > 0.05; Figure 2f). At the lowest feed dose-to-larval density (0.4 mg VS larva^–1^ cm^–2^) tested, the frass obtained from both substrates appeared visually more dust-like compared to frass obtained at 2.6 mg VS larva^–1^ cm^–2^.

### 3.3 Pathogen dynamics and inactivation

#### 3.3.1 Experiment 2: single substrate inoculation day

Pathogen reduction was observed from Day 3 and progressed until Day 14, when the experiment was terminated (Figures 3a, b). For *Salmonella* spp., the initial inoculum concentration was about 8 log_10_ CFU g^−1^ in both substrates. By Day 6, *Salmonella* spp. population had reduced to about 6 log_10_ CFU g^−1^ and 5 - 6 log_10_ CFU g^−1^ in the frass of the carbohydrate-rich and protein-rich substrates, respectively (Figure 3a). By Day 10, *Salmonella* spp. population had further reduced reaching about 4 - 6 log_10_ and 3 - 4 log_10_ in frass of the carbohydrate-rich and protein-rich substrate, respectively (Figure 3a). The total pathogen reduction (Δlog_10_ Red) from Day 1 to Day 14 was calculated to determine the impact of the treatments on pathogen inactivation (Figure 3c and Table 2).

**FIGURE 3:**
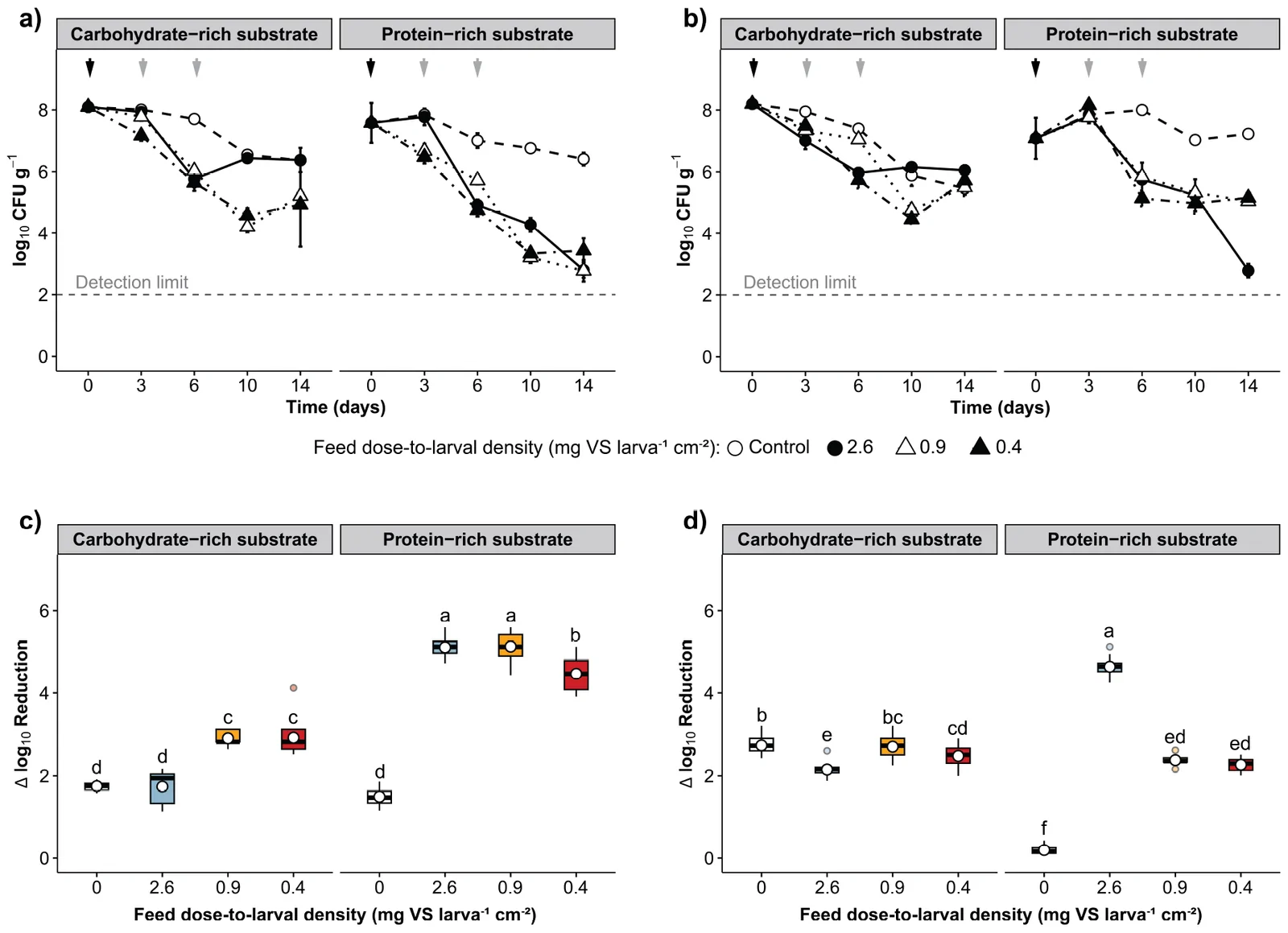
Pathogen dynamics (log_10_ CFU g^–1^) and reduction (Δlog_10_ Reduction) of bacterial pathogens during bioconversion of a carbohydrate-rich and protein-rich substrate using black soldier fly, *Hermetia illucens* larvae (BSFL) in Experiment 2. Pathogen dynamics of *Salmonella* spp. **(a)** and *Escherichia coli* **(b)**; total logarithmic pathogen reduction of *Salmonella* spp. **(c)** and *E. coli* **(d)** in treatment residues. Larvae were fed pathogen-inoculated substrates only on the first feeding event (indicated by black arrowheads), followed by non-inoculated substrates in the subsequent two feeding events (indicated by grey arrowheads). All values represent mean ± standard deviation. Boxes **(c,d)** display interquartile range (IQR), median (bold horizontal line inside each box), mean (white circle), and the range of data represented by whiskers (vertical lines). Data points farther than 1.5 × IQR from boxes denote outliers. Different letters indicate statistically significant differences among treatments.

**TABLE 2:** Summary results of inactivation constant (*k*) and decimal reduction time (D_90_) for *Salmonella* spp. and *Escherichia coli* after bioconversion of the carbohydrate-rich and protein- rich substrates using black soldier fly, *Hermetia illucens* larvae (BSFL) in Experiments 2 and 3.

Substrate	Feed load-to-larval density (mg VS larva^–1^ cm^–2^)	Single substrate inoculation				Multiple substrate inoculations			
		*k* *Salmonella* spp.^§^ (log_10_ day^–1^)	D_90_ (days) *Salmonella* spp.^‡^	*k* *E. coli*^‡^ (log_10_ day^–1^)	D_90_ (days) *E. coli*^‡^	*k* *Salmonella* spp.^¶^ (log_10_ day^–1^)	D_90_ (days) *Salmonella* spp.^¶^	*k* *E. coli*^‡^ (log_10_ day^–1^)	D_90_ (days) *E. coli*^‡^
Carbohydrate-rich	0	−0.13 ± 0.01^d^	8.0 ± 0.5	−0.20 ± 0.01	5.2 ± 0.4	−0.05 ± 0.01	21.1 ± 3.9	−0.06 ± 0.01	16.2 ± 1.3
	2.6	−0.12 ± 0.03^d^	8.5 ± 2.2	−0.15 ± 0.01	6.5 ± 0.5	–	–	−0.03 ± 0.01	41.0 ± 15.6
	0.9	−0.21 ± 0.01^c^	4.8 ± 0.3	−0.19 ± 0.02	5.2 ± 0.5	−0.06 ± 0.01	17.2 ± 2.1	−0.08 ± 0.01	12.8 ± 2.4
	0.4	−0.21 ± 0.03^c^	4.9 ± 0.5	−0.18 ± 0.02	5.7 ± 0.7	−0.06 ± 0.01	15.6 ± 1.9	−0.06 ± 0.01	16.1 ± 1.9
Protein-rich	0	−0.11 ± 0.01^d^	9.6 ± 1.3	−0.01 ± 0.01	94.2 ± 54.3	–	–	−0.04 ± 0.02	36.9 ± 18.7
	2.6	−0.37 ± 0.02^a^	2.7 ± 0.1	−0.33 ± 0.01	3.0 ± 0.1	−0.12 ± 0.01	8.1 ± 0.3	−0.11 ± 0.01	9.0 ± 1.0
	0.9	−0.37 ± 0.03^a^	2.7 ± 0.2	−0.17 ± 0.01	5.9 ± 0.3	–	–	−0.06 ± 0.02	17.4 ± 5.1
	0.4	−0.32 ± 0.03^b^	3.2 ± 0.3	^–^0.16 ± 0.01	6.2 ± 0.5	–	–	−0.07 ± 0.01	15.2 ± 1.7
Statistics									
Larval density		*F*_3,112_ = 373.6 *P* < 0.001	*H*_3_ = 55.7 *P* < 0.001	*H*_3_ = 10.5 *P* = 0.015	*H*_3_ = 10.5 *P* = 0.015	–	–	*H*_3_ = 10.4 *P* = 0.016	*H*_3_ = 10.4 *P* = 0.016
Substrate		*F*_1,112_ = 963.2 *P* < 0.001	*H*_1_ = 27.5 *P* < 0.001	*H*_1_ = 2.9 *P* = 0.088	*H*_1_ = 2.9 *P* = 0.088	–	–	*H*_1_ = 2.9 *P* = 0.086	*H*_1_ = 2.9 *P* = 0.086
Feed dose-to-larval density: Substrate		*F*_3,112_ = 187.1 *P* < 0.001	*H*_3_ = 25.5 *P* < 0.001	*H*_3_ = 82.5 *P* < 0.001	*H*_3_ = 82.5 *P* < 0.001	–	–	*H*_3_ = 64.7 *P* < 0.001	*H*_3_ = 64.7 *P* < 0.001

BSFL were fed pathogen-inoculated substrates only on the first feeding event (i.e., single substrate inoculation), followed by non-inoculated substrates in the subsequent two feeding events in Experiment 2. In Experiment 3, BSFL were fed pathogen-inoculated substrates on all the three feeding events (i.e., multiple substrate inoculations). Values represent mean ± standard deviation. ^§^ Within each column, different lowercase letters indicate significant differences among groups (One-way ANOVA, with Tukey’s *post hoc* test, *P* < 0.05). *P* < 0.05, *P* < 0.01, *P* < 0.001. ^‡^Data tested using the Scheirer-Ray-Hare test (an extension of the Kruskal-Wallis test) followed by Dunn’s *post hoc*; ^¶^ Data was not tested statistically as no reduction was observed in most treatments.

The Δlog_10_ Red of *Salmonella* spp. was significantly impacted by feed dose-to-larval density (*F*_3,112_ = 373.6, *P* < 0.001) and substrate type (*F*_1,112_ = 963.2, *P* < 0.001; Figure 3c). Moreover, a significant interaction effect on Δlog_10_ Red of *Salmonella* spp. was observed between feed dose-to-larval density and substrate type (*F*_3,112_ = 187.1, *P* < 0.001). Notably, the highest Δlog_10_ Red of *Salmonella* spp. was achieved in the protein-rich substrate, particularly at 2.6 and 0.9 mg VS larva^–1^ cm^–2^. At 2.6 and 0.9 mg VS larva^–1^ cm^–2^, the Δlog_10_ Red of *Salmonella* spp. was 5.1, but declined to 4.5 at 0.4 mg VS larva^–1^ cm^–2^, compared to 1.5 in the control (Figure 3c). By contrast, the Δlog_10_ Red of *Salmonella* spp. in the carbohydrate-rich substrate was 1.7, 2.9 and 2.9 at feed dose-to-larval densities of 2.6, 0.9 and 0.4 mg VS larva^–1^ cm^–2^, compared to 1.8 in the controls (Figure 3c).

For *E. coli*, the initial inoculum concentration was about 8 log_10_ CFU g^−1^ in both substrates. By Day 6, *E. coli* population had reduced to about 6 - 7 log_10_ CFU g^–1^ and 5 - 6 log_10_ CFU g^–1^ in residues of carbohydrate-rich and protein-rich substrate, respectively (Figure 3b). By Day 10, *E. coli* population had further reduced reaching about 5 - 6 log_10_ and 5 log_10_ in residues of carbohydrate-rich and protein-rich substrates, respectively (Figure 3b). The Δlog_10_ Red of *E. coli*, was significantly impacted by feed dose-to-larval density (*F*_3,112_ = 461.4, *P* < 0.001) and substrate (*F*_1,112_ = 15.5, *P* < 0.001; Figure 3d). Additionally, a significant interaction effect on Δlog_10_ Red of *E. coli* was observed between larval density and substrate type (*F*_3,112_ = 780.7, *P* < 0.001).

Notably, the highest Δlog_10_ Red of *E. coli* achieved was 4.6 during 14 days in the protein-rich substrate, where BSFL received 2.6 mg VS larva^–1^ cm^–2^. However, decreasing the feed dose-to-larval density to 0.9 and 0.4 mg VS larva^–1^ cm^–2^ reduced the Δlog_10_ Red of *E. coli* to 2.4 and 2.3, respectively, compared to 0.2 in the control (Figure 3d). By contrast, the Δlog_10_ Red of *E. coli* in the carbohydrate-rich substrate was 2.2, 2.7 and 2.5 at feed dose-to-larval densities of 2.6, 0.9 and 0.4 mg VS larva^–1^ cm^–2^. However, a 2.7 Δlog_10_ Red of *E. coli* was observed in the controls (Figure 3d).

#### 3.3.2 Experiment 3: multiple substrate inoculations

For *Salmonella* spp., pathogen reduction did not differ significantly from that in the control at a feed dose-to-larva density of 2.6 mg VS larva^–1^ cm^–2^ in the carbohydrate substrate, 0.9 and 0.4 mg VS larva^–1^ cm^–2^ in the protein-rich substrate (Figures 4a, b). Thus, the Δlog_10_ Red data for *Salmonella* spp. was not tested statistically. Similar to single substrate inoculation (Experiment 2), we found that pathogen reduction declined with decreasing feed dose-to-larval density (Figure 4 and Table 2). Notably, however, pathogen reduction was overall lower in multiple substrate inoculations than single substrate inoculation.

**FIGURE 4:**
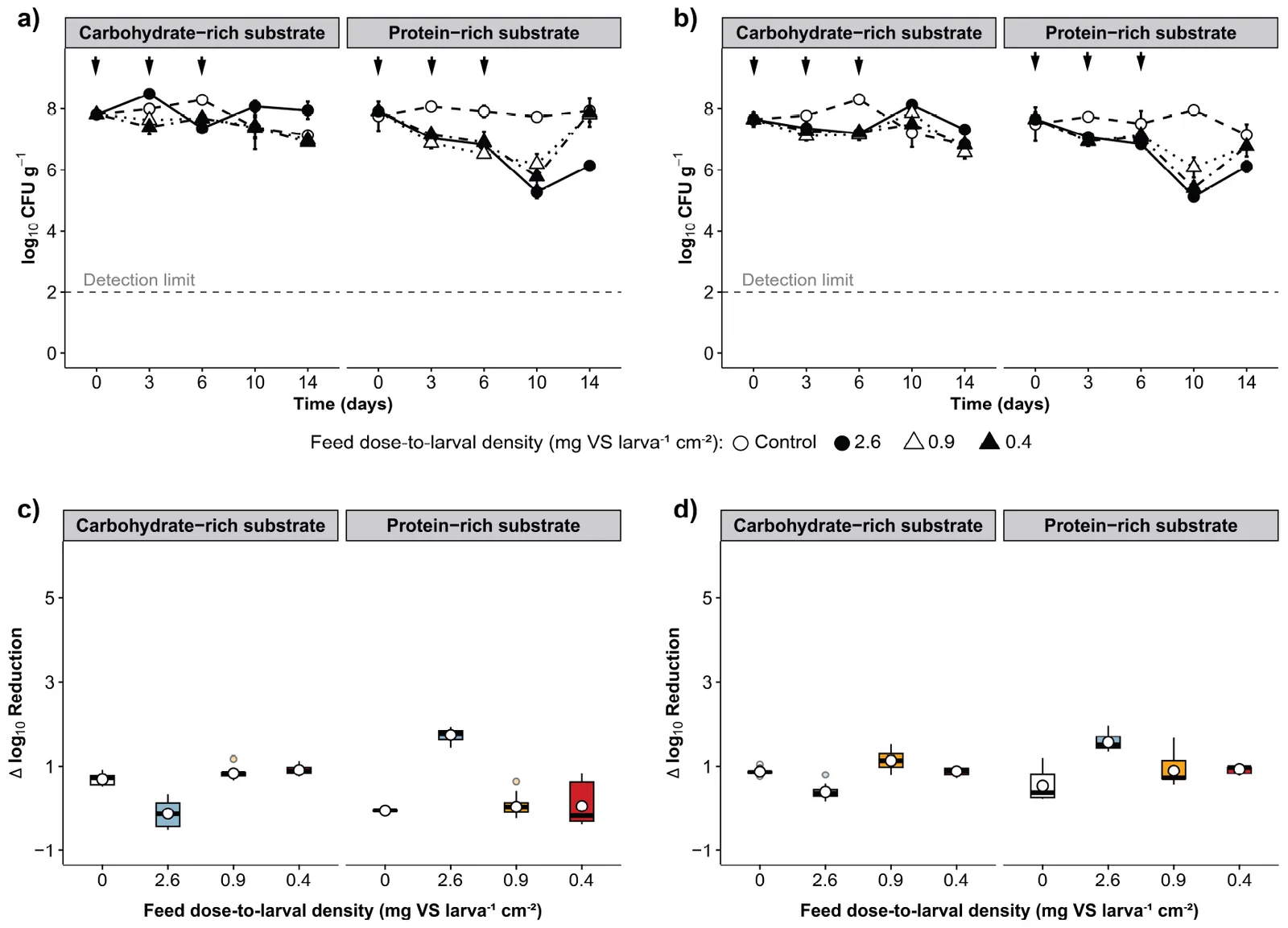
Pathogen dynamics (log_10_ CFU g^–1^) and reduction (Δlog_10_ Reduction) of bacterial pathogens during bioconversion of a carbohydrate-rich and protein-rich substrate using black soldier fly, *Hermetia illucens* larvae (BSFL) in Experiment 3. Pathogen dynamics of *Salmonella* spp. **(a)** and *Escherichia coli* (**b**); total logarithmic pathogen reduction of *Salmonella* spp. **(c)** and *E. coli* (**d**) in treatment residues. Larvae were fed pathogen-inoculated substrates on all three feeding events (indicated by black arrowheads). All values represent mean ± standard deviation. Boxes (**c,d**) display interquartile range (IQR), median (bold horizontal line inside each box), mean (white circle), and the range of data represented by whiskers (vertical lines). Data points farther than 1.5 × IQR from boxes denote outliers.

## 4 Discussion

### 4.1 Experiment 1: process efficiency

Bioconversion efficiency increased with larval density but was higher in the carbohydrate-rich than protein-rich substrate (Figure 2a) which is consistent with previous studies on BSFL (Lopes et al., 2023; Klammsteiner et al., 2025). Klammsteiner et al. (2025) noted that increasing the larval density from 1.25 to 5 larvae cm^–2^ increased bioconversion efficiency nearly two-fold. In the study of Klammsteiner et al. (2025), the feed load was 0.1 g of substrate (food waste) larva^–1^ day^–1^. A possible explanation for our findings is that BSFL increased their food intake (compensatory feeding) and/or metabolism when reared at low feed dose-to- density on the carbohydrate-rich (low-protein) substrate, to meet the larvae their protein requirements.

Insects actively adjust their food intake according to physiological needs (Lee, 2017; Rho and Lee, 2023). This enables insects to ameliorate the negative consequences of low nutrient availability in the food (Lee, 2017; Rho and Lee, 2023). Additionally, insects can increase post-ingestive utilization efficiency of a specific nutrient if ingested in deficit relative to the required level. For example, insects including BSFL homeostatically compensate for a lack of energy by increasing their food intake or consumption rate and modification of post-ingestive utilization (Colasurdo et al., 2009; Barragán-Fonseca et al., 2018; Gold et al., 2020; Chen et al., 2023). BSFL require a substrate containing a balanced composition of macro- and micronutrients for optimal growth (Rehman et al., 2017; Lalander et al., 2019; Barragán-Fonseca et al., 2021). Berggreen et al. (2025) reported that 19.4 % dietary protein with a corresponding protein-to-carbohydrate ratio of 1:2.8, is optimal for maximizing larval dry-mass production. However, protein quality is determined by digestibility and the balance of amino acid profiles (Millward, 2012). In eukaryotes, proteins are assembled from 20 amino acids, nine of which are considered essential and must be supplied in the diet (Millward, 2012; Table 1). Berggreen et al. (2025) reported that excluding any of the essential amino acids (lysine, isoleucine, leucine, phenylalanine, threonine, tryptophan, valine or arginine) in the substrate led to reduced BSFL growth. In our study, the individual concentration of most amino acids was two- to threefold higher in the protein-rich diet than the carbohydrate-rich substrate (Table 1). However, the specific contribution of individual amino acids to larval performance remain unclear.

Altogether, these findings suggest that feed dose-to-larval density and the physico-chemical properties of the substrate (e.g., macronutrients) impact process efficiency. Consistent with this premise, we found that bioconversion efficiency and substrate reduction were higher in the carbohydrate-rich than the protein-rich substrate at feed dose-to-larval densities 0.9 and 0.4 mg VS larva^–1^ cm^–2^ (Figures 2a,e), which suggests compensatory feeding. Gold et al. (2020) showed that BSFL adjust their food ingestion, enzyme production and absorption to meet nutrient demands, when reared on low-protein (carbohydrate-rich) substrates. Thus, in our study, the increase in bioconversion efficiency with decreasing feed dose-to-larval density can be attributed to compensatory feeding and/or increased metabolism.

During bioconversion, substrate reduction is largely attributed to larval activity and the activity of BSFL-associated microbial communities including bacteria, fungi and archaea (Fuhrmann et al., 2025; Klammsteiner et al., 2025; Gurusinga et al., 2026). Accordingly, factors that impact larval activity and/or the activity and structure of BSFL-associated microbial communities could synergistically impact process efficiency. For example, Kreft et al. (2026) revealed that substrate type was the dominant factor influencing frass-associated microbiota. Specifically, high-fibre diets promoted the abundance and diversity of fungi, while high-protein substrates enriched specific bacterial taxa (Kreft et al., 2026). Another possible factor is ammonia, which is produced during BSFL bioconversion (Coudron et al., 2024). Ammonia is governed by the ammonium-ammonia (NH_3_ : NH_4_^+^) equilibrium, which in turn depends on the pH and temperature (Nordin et al., 2010). Uncharged ammonia (NH_3_) is toxic to microorganisms, and its concentration rises with temperature (Nordin et al., 2010). However, as we did not measure temperature or ammonia concentrations in our study, we cannot confidently attribute these to the treatments in our study, and we acknowledge this as a limitation. Taken together, in our study, low feed dose-to- larval density could have impacted BSFL and the activity of BSFL-associated microorganisms, thereby enhancing substrate reduction.

Final larval weight decreased with decreasing feed dose-to-larval density, regardless of substrate type (Figures 2c,d; Supplementary Figure 1). This finding suggests that increasing larval density while maintaining the same amount of substrate (*i.e.*, low feed dose-to-larval density), reduces BSFL growth (the increase in body size and progression through life stages). This finding is consistent with previous studies, but the underlying mechanisms are unclear (Barragán-Fonseca et al., 2018; Jones and Tomberlin, 2019; Schreven et al., 2022; Lopes et al., 2023; Muurmann et al., 2025). In our study, BSFL received the same amount of substrate, which resulted in a feed dose-to-larval density of 2.6, 0.9 and 0.4 mg VS larva^–1^ cm^–2^. Thus, it is likely that the low feed dose at high larval density could have resulted in limited access to resources (particularly nutrients) and a concomitant accumulation of metabolic waste products such as ammonia, which could have stressed the larvae and subsequently reduced larval growth (Barragán-Fonseca et al., 2018; Lu and Wang, 2025). Thus, it would be interesting to assess the impact of larval density by allocating BSFL the same amount of feed dose (*i.e.*, same feed dose-to-larval density). However, this could have additional negative consequences, such as such as excessive substrate depth (Lopes et al., 2023).

Reduced larval growth could also, in part, be attributed to elevated temperature as have been observed to occur in decomposing substrate at high larval densities (Belperio et al., 2024; Klammsteiner et al., 2025). For example, Klammsteiner et al. (2025), noted that doubling the larval density during bioconversion of food waste resulted in increased temperatures by up to 2.4 °C in the decomposing substrate. Growth restriction at high temperature is a widespread phenomenon among ectothermic animals including insects (Ghosh et al., 2013). This phenomenon, termed the inverse temperature size rule, describes the general observation that ectothermic species develop faster with increasing temperature, but become smaller in body size (Atkinson, 1994; Ghosh et al., 2013; Texada et al., 2020). Thus, while ambient temperature promotes BSFL development (Chia et al., 2018), increase in larval density could elevate substrate temperature well above ambient temperature levels. This, in turn, could exert physiological stress on BSFL (Opare et al., 2023) and thereby restrict larval growth.

Substrate reduction increased significantly with decreasing feed dose-to-larval density and was higher in the carbohydrate-rich than the protein- rich substrate (Figure 2e). Consequently, frass yield declined with decreasing feed dose-to-larval density and was higher in the protein-rich than carbohydrate- rich substrate (Figure 2f). These findings, suggest that feed dose-to-larval density and the physico-chemical properties of the substrate, such as macronutrients (Table 1) profoundly influence substrate reduction and frass yield. Moreover, increased substrate reduction and a concomitant decline in frass yield may also be attributed to compensatory feeding and/or increased metabolism.

### 4.2 Experiment 2: single substrate inoculation

Consistent with our hypothesis, we found that pathogen reduction was higher in the protein-rich than the carbohydrate-rich substrate (Figure 3). Unexpectedly, however, we also found that pathogen inactivation declined with decreasing feed dose-to-larval density, which was contrary to our second hypothesis. More broadly, this unexpected finding suggests that pathogen inactivation in BSFL bioconversion is strongly influenced by feed dose-to-larval density. Additionally, because BSFL received progressively lower volatile solids (VS) at high density, it seems probable that pathogen inactivation in BSFL bioconversion is also linked to VS availability. VS is a coarse proxy for the total organic matter, *i.e.* macronutrients such as the composition of protein, fat and carbohydrate in the substrates.

Liu et al. (2023) found that BSFL preferentially utilize carbohydrates as the primary substrate for fatty acid biosynthesis, only switching to dietary fat once carbohydrate reserves are depleted. Notably, the substrate fat content in the present study differed markedly from that in a previous study (Lalander et al., 2025), where *Salmonella* spp. inactivation was observed at similar larval VS feed dose (0.25 g VS larva^–1^) and larval density (5 larvae cm^–2^). Compellingly, the feed dose-to-larval density in Lalander et al. (2025) and Lopes et al. (2024) corresponds to 0.1 mg VS larva^–1^ cm^–2^, which is four-fold lower than the minimum (0.4 mg VS larva^–1^ cm^–2^) tested in the present study. Moreover, the substrate (food waste) used in Lopes et al. (2024) and contained less protein (19.1 % of DM) compared to the protein-rich substrate in the present study (32 % DM). However, the food waste contained substantially more fat (26.7 % DM) compared to the protein-rich substrate in the present study (11.6 % DM) (Lopes et al., 2024). Thus, despite its high protein content, the relatively low-fat content in the protein-rich substrate in the present study may have caused rapid depletion of available energy reserves at high larval density, potentially limiting the metabolic resources available for BSFL immune responses.

Pathogen reduction in BSFL bioconversion has, in part, been linked to BSFL immune responses particularly the biosynthesis of antimicrobial substances such as AMPs (Lalander et al., 2025). AMPs are small proteins with potent antimicrobial activity against various microorganisms including bacteria. The BSF genome encodes about 50 AMP genes (*e.g.*, cecropins, attacins and defensins) and is the second largest AMP repertoire reported among insects (Moretta et al., 2020; Zhan et al., 2020; Szczepanik and Świątkiewicz, 2024). AMP biosynthesis in BSFL can be influenced by pathogen exposure, age of larvae and substrate composition (Vogel et al., 2018; Sun et al., 2023; Fahmy et al., 2024; Herman et al., 2024; Giglio et al., 2025). For instance, using transcriptome analysis, Vogel et al. (2018) reported higher expression of AMP-encoding genes in BSFL reared on protein-rich substrates or supplemented with sunflower oil. This suggests that dietary protein, as well as fat, may modulate immune responses, although the mechanisms remain unclear. Similarly, Candian et al. (2023) reported lower production of AMPs and hemocytes (insect blood cells) in BSFL reared on diets with a low protein-to-carbohydrate ratio. Conceivably, the protein-rich substrate in our study could, in part, could have enhanced pathogen inactivation via an AMP-dependent mechanism. However, substrate depletion at low feed dose-to-larval density may have constrained the larval immune capacity, thereby limiting AMP-mediated pathogen inactivation.

In various insects, high rearing density is associated with upregulation of immune responses, including the biosynthesis of AMPs, a phenomenon termed density-dependent prophylaxis (Hughes et al., 2002; Cotter et al., 2004). However, to the best of our knowledge, AMPs have not been isolated from BSFL frass, and the role of AMPs in pathogen inactivation during BSFL bioconversion remains unclear. Thus, we attempted to identify AMPs in BSFL frass and fat tissues in a pre-test experiment. To do this, we analyzed frass and fat tissues from BSFL reared on the carbohydrate-rich and protein-rich substrate. Then, targeted analysis of attacins and cecropins was conducted using liquid chromatography-tandem mass spectrometry (LC-MS/MS)-based proteomics. We detected AMPs in fat tissues of larvae reared on both substrates, but no AMPs were detected in the frass (Supplementary Table 1). Specifically, three attacins were detected in fat tissues of BSFL reared on the protein-rich substrate, while two attacins were detected in the fat tissues of larvae reared in the carbohydrate-rich substrate. Cecropins were not detected in any of the samples (Supplementary Table 1). Our inability to detect AMPs in frass was largely attributed to the complexity of the frass matrix. This, in part, could have resulted in extraction inefficiencies. Secondly, we were unable to perform absolute quantification of AMPs in the samples as this requires stable isotope-labelled internal standards (*e.g*., ^13^C or ^15^N- labelled version of the target AMPs) which are currently not commercially available for BSFL. Nonetheless, these results suggest that a protein-rich substrate may be associated with changes in larval immune responses, potentially including AMP biosynthesis. However, our findings do not establish a direct link between substrate composition, AMP production and pathogen inactivation during bioconversion.

### 4.3 Experiment 3: multiple substrate inoculations

In Experiment 3, BSFL were fed pathogen-inoculated substrates on all three feeding events, *i.e*. multiple substrate inoculations (Figure 1). Similar to single substrate inoculation (Experiment 2), we found that pathogen reduction declined with decreasing feed dose-to-larval density particularly in the protein-rich substrate (Figure 4). Notably, however, pathogen reduction was generally low with multiple substrate inoculations when compared to single substrate inoculation (Figures 3, 4). Moreover, no reduction of *Salmonella* spp. was observed in treatments with 0.9 and 0.4 mg VS larva^–1^ cm^–2^ (Figure 4). More broadly, these findings suggest that pathogen inactivation in BSFL bioconversion strongly depends on the frequency of pathogen inoculation in to the decomposing substrate. In single substrate and multiple substrate inoculations, the initial pathogen inoculum was about 8 log_10_ CFU g^–1^ in both the carbohydrate-rich and protein-rich substrate. However, multiple substrate inoculations could have resulted in a booster inoculum effect, thereby allowing build-up of pathogen load in the decomposing substrate.

Consistent with this, Lopes et al. (2020) showed that BSFL bioconversion of pathogen-inoculated bread waste-aquaculture waste mixture supplied in three feeding events achieved only a 3.4 log_10_ reduction in *Salmonella* spp. However, a nearly two-fold greater log_10_ reduction was obtained when the same substrate was supplied as lump sum (*i.e.*, single feeding event) or supplied in three feeding events with a single inoculation. A similar pattern was observed for *E. coli* (Lopes et al., 2020). The difference in treatment outcomes was attributed to addition of contaminated substrate more than once, which presumably may have resulted in increased pathogen load. Conversely, Lalander et al. (2025) reported similarly high reductions in *Salmonella* spp. and *E. coli* during the bioconversion of pathogen-inoculated food waste. Pathogen reduction was primarily attributed to elevated temperature in the decomposing substrate, which is typically higher for decomposing substrates in the larger rearing trays than smaller ones (Yakti et al., 2022). Together, these findings suggest that larval density and rearing scale influence substrate temperature and may consequently affect bioconversion efficiency and the microbiological safety of the process and its products. Therefore, it will be important to monitor substrate temperature during bioconversion.

In our study, pathogen reduction was limited or absent at low feed dose-to-larval density, suggesting that the pathogens were unaffected by BSFL treatment in multiple substrate inoculations (Experiment 3). Our findings are consistent with Lopes et al. (2020), who showed that BSFL treatment reduced *Salmonella* spp. and *E. coli* ATCC 13706 during bioconversion of aquaculture waste-bread mixture particularly when the substrate was inoculated only at the first feeding event. Altogether, our findings show that BSFL bioconversion achieves limited pathogen reduction and may be ineffective if the decomposing substrate contains elevated pathogen load, as observed in multiple substrate inoculations. However, such a high pathogen load (8 log_10_ CFU g^–1^) for *Salmonella* spp. and *E. coli* is rarely encountered in biowaste (Hutchison et al., 2005; Robertson et al., 2010; Caro et al., 2023). Another possible consideration to enhance pathogen reduction would be to consider a single feeding event (Lopes et al., 2020).

Notably, pathogen reduction observed in the control, particularly in the carbohydrate-rich substrate, likely reflects anaerobic degradation arising from the absence of continuous mixing in the controls, in contrast to larval treatments. Under anaerobic conditions, natural microbial activity especially by fermentative bacteria such as such as *Bacteroides* spp. can promote the production of short-chain fatty acids (SCFAs), including butyrate and propionate (Jacobson et al., 2018). These SCFAs have been shown to inhibit enteric pathogens such as *Salmonella* spp. by lowering pH, and disruption of membrane function (Rinzema et al., 1994; Jacobson et al., 2018; Hockenberry et al., 2021).

## 5 Conclusion

Previous studies have shown that BSFL bioconversion can inactivate notorious food-borne pathogens such as *Salmonella* spp. and *E. coli* in biowaste substrates. However, how factors such as substrate type, feed dose, and larval density impact pathogen reduction is unclear. Our study confirms and extends previous findings by showing that process efficiency and pathogen inactivation in BSFL bioconversion are inextricably linked. Moreover, the effects of larval density and feed dose are inherently linked. Increasing larval density without adjusting the feed dose alters the feed dose per larva. To account for this limitation, we introduce a new parameter termed, *feed-dose-to-larval density*, which captures a density- and area-adjusted feed dose. Regarding process efficiency, bioconversion efficiency, larval yield and substrate reduction increased with decreasing feed dose-to-larval density. Additionally, larval survival, final larval weight and frass yield declined with decreasing feed dose-to-larval density. In contrast, pathogen reduction declined with decreasing feed dose-to-larval density but was higher in the protein-rich than carbohydrate-rich substrate. Moreover, pathogen reduction was limited or absent in multiple substrate inoculations compared to single substrate inoculation. Thus, a low feed dose-to- larval density could enhance some process efficiency parameters but compromise the microbiological hygiene and safety of the process and products. Collectively, our findings underscore the need for integrated optimization of process efficiency and microbiological hygiene in BSFL bioconversion.

## Data Availability

The original contributions presented in this study are included in this article/Supplementary material, further inquiries can be directed to the corresponding author.
