# Supplementary material for: Low feed dose-to-larval density attenuates pathogen inactivation in black soldier fly larvae bioconversion
Source: Front Microbiol. 2026 Sep 23;17:1909800. doi: 10.3389/fmicb.2026.1909800 (PMC13679362; doi:10.3389/fmicb.2026.1909800)
Supplement: Supplementary file 1 [file Data_Sheet_1.pdf]

Supplementary material

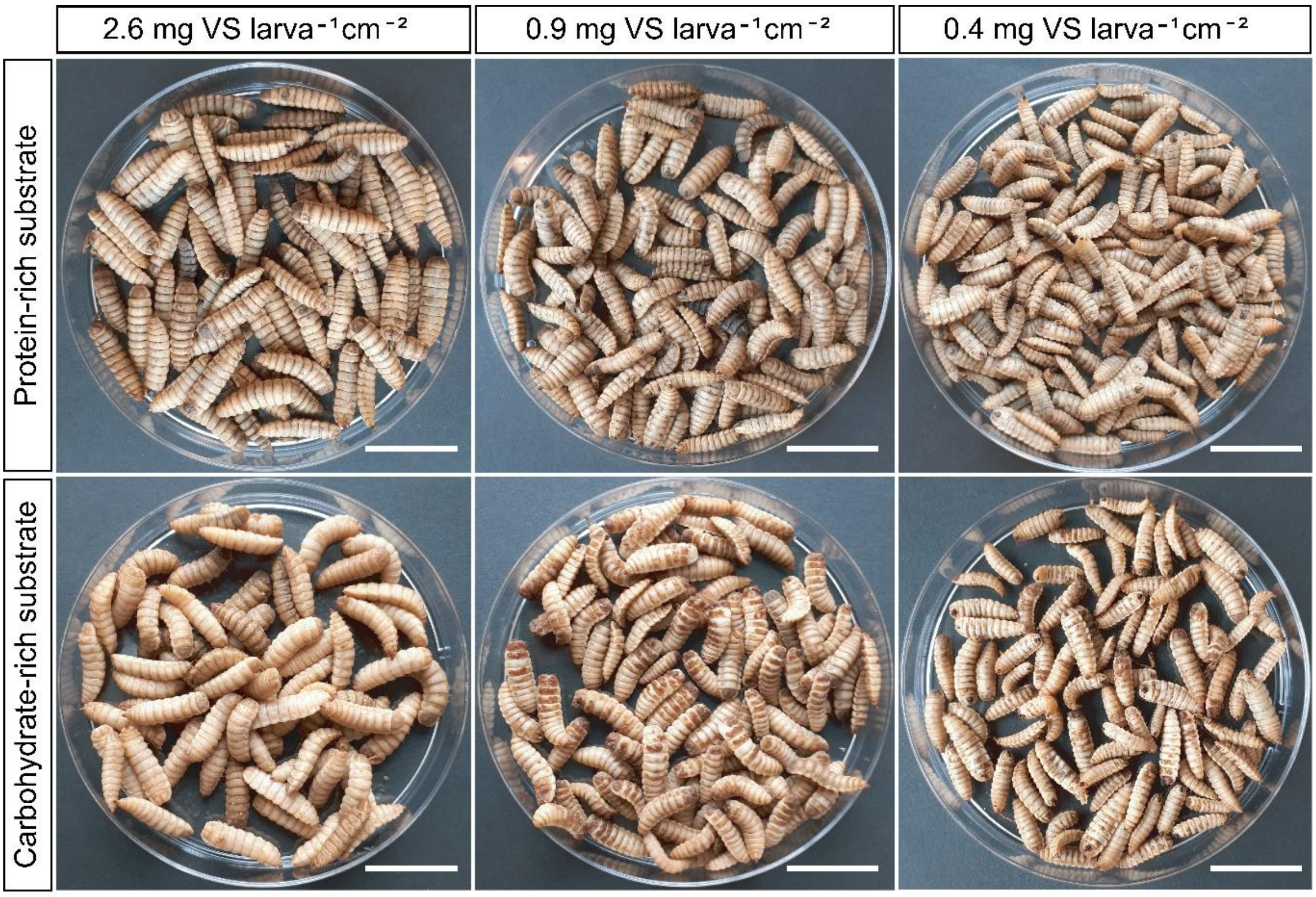

Figure S1: Images of black soldier fly, Hermetia illucens (BSFL) larvae reared at different feed dose-to-larval density (2.6, 1.3, and 0.4 mg VS larva^{-1} cm^{-2}) on the carbohydrate-rich and protein-rich substrate. Scale bar ≈ 20 mm

Table S1: Antimicrobial peptides (attacins) detected in fat tissues of black soldier fly larvae reared on the carbohydrate-rich and protein-rich substrate. Table shows the amino acid sequences, molecular weight (in kDa), and Mascot/identification score of the detected attacins.

Substrate	AMP detected	Sequence	MW [kDa]	Score
Carbohydrate-rich	Hill_BB_C16882_attactin	LnnGFAFDKDHSLGVSKTITPGVSDTFSqNAR	18.6	1.6
	Hill_BB_C10074_attactin	RGSASVTVNNESRSPQDGRRSSLDAnAYYQR	16.7	1.6
Protein-rich	Hill_BB_C16883_attactin	FLGNPNHnIGGGVFASGNTRAnLWKSPSGLSTFDLTGSAS	18.6	1.7
	Hill_BB_C16883_attactin	TPDHRVDANVFnSHTRLnNGFAFDKSNTPSLGAFGTLnLKDHSLGVSK	18.6	1.5
	Hill_BB_C16882_attactin	SNTPSLGAFGTLnLKDHSLGVSKRGGSLDYTHRAGHALSLGASHIPK	18.6	1.5
